# Transcriptional profiling of *Auricularia cornea* in selenium accumulation

**DOI:** 10.1038/s41598-019-42157-2

**Published:** 2019-04-04

**Authors:** Xiaolin Li, Lijuan Yan, Qiang Li, Hao Tan, Jie Zhou, Renyun Miao, Lei Ye, Weihong Peng, Xiaoping Zhang, Wei Tan, Bo Zhang

**Affiliations:** 1 Soil and Fertilizer Institute, Sichuan Academy of Agricultural Sciences, Chengdu, 610066 China; 2 Chair for Aquatic Geomicrobiology, Institute of Biodiversity, Friedrich Schiller University Jena, Jena, D-07743 Germany; 3 Research Center of Edible Fungi, Sichuan Academy of Agricultural Sciences, Chengdu, 610066 China; 4 College of Life Science, Sichuan University, Chengdu, 610065 China; 5 Department of Microbiology, College of Resources, Sichuan Agricultural University, Chengdu, 611130 China

## Abstract

*Auricularia cornea* is a widely cultivated edible fungus with substantial nutritive value. This study aimed to enrich the multifunctional bionutrient element selenium in *A. cornea* to improve its quality and explore the accumulation of selenium in the fungus using high-throughput RNA-Seq technology. In general, the treatment group with a 100 µg/g supply of selenium outperformed the other treatment groups in terms of high yield, rich crude polysaccharides and a high total selenium concentration. Additional evidences demonstrated the budding and mature phases were two typical growth stages of *A. cornea* and were important for the accumulation of selenium. Therefore, the budding and mature phase tissues of *A. cornea* in the treatment group with a 100 µg/g supply of selenium were used for transcriptome analysis and compared to those of a control group that lacked additional selenium. A total of 2.56 × 105 unigenes from *A. cornea* transcriptome were assembled and annotated to five frequently used databases including NR, GO, KEGG, eggNOG and SwissProt. GO and KEGG pathway analysis revealed that genes involved in metabolic process and translation were up-expressed at the budding stage in response to selenium supplementation, including amino acid metabolism, lipid metabolism, ribosome. In addition, the differential gene expression patterns of *A. cornea* suggested that the up-expressed genes were more likely to be detected at the budding stage than at the mature stage. These results provide insights into the transcriptional response of *A. cornea* to selenium accumulation.

## Introduction

*Auricularia cornea* (synonym: *A. polytricha*) belongs to the *Basidiomycota* and is an edible fungus with high nutritive value because it is rich in amino acids, polysaccharides, vitamins and mineral substances in its fruiting bodies^1–3^. Due to its significant medicinal value and high economic returns, the cultivation area of *A. cornea* is constantly increasing^4^. The yield of *A. cornea* is stable with sophisticated cultivation skills. Currently, substantial efforts are underway to improve its quality and growth efficiency to meet the increased market demands.

As a multifunctional bionutrient element, selenium was identified as one of the necessary trace elements for the human body by World Health Organization (WHO) Committee in 1973^5^ and was listed as an essential element for daily diets by the Chinese Nutrition Society in 1988^6^. In addition, selenium is an important component of glutathione peroxidases and selenoproteins, which are important to maintain human health because of their antioxidative abilities, antitumour effects, ability to strengthen immunity and aid in the control of various diseases, including cancer and heart disease^7–9^.

Since selenium is found in low concentrations in the soil, plants and edible mushrooms, such as *Flammulina velutipes*, *Pleurotus ostreatus* and *Ganoderma lucidum*, organisms that can accumulate selenium are an ideal selenium-rich food^10–14^. Since mushrooms can biotransform selenium from inorganic to organic forms, selenium enrichment studies in mushrooms are usually performed by adding inorganic selenium to the culture medium. For instance, the addition of sodium selenite to the substrate increased the uptake and integration of selenium into proteins, polysaccharides and other components in *G. lucidum*^15^. The quality and nutritive value of edible mushrooms are generally improved by adding essential elements, such as selenium and calcium, to the substrate via irrigation water^16^. The addition of selenium to the substrate has a substantial impact on the growth and development of the fruiting bodies of *A. cornea* in direct and indirect ways, such as changing its crude polysaccharide and trace element concentrations and affecting the enzyme activities and microbial diversity in the substrate^17–20^. As a transformation carrier of mineral elements^21^, *A. cornea* can be cultivated in substrate supplied with inorganic selenium to harvest the fruiting bodies that may have a nutritive value beyond their original state. However, *A. cornea* can withstand a high range of selenium concentrations in cultivation, so it is critical to identify the optimal concentration of selenium^22,23^.

Currently, next-generation sequencing (NGS) is widely utilized to investigate complex genomic questions with unprecedented handling capacity, scalability and speed^24^. Transcriptome sequencing explores all the mRNAs, gene composition and functions in a given sample. Comparative transcriptome analysis provides insights into the differential expression patterns of genes and related biological pathways in cells, tissues or individuals during different physiological conditions^25,26^. For example, Yu *et al*.^27^ studied the changes in gene expression and biological pathways from the mycelia to the initial primordial stages of *G. lucidum* using comparative transcriptome analysis. In addition, RT-PCR technology has been widely used in the quantitative detection of genes and viruses due to its high speed, sensitivity and accuracy^28–30^. For instance, real-time quantitative PCR was used to study the stability of housekeeping genes in the symbiotic fungus *Tuber melanosporum* during its development and discovered various genes that were stably expressed at different stages^31^.

Although selenium enrichment in edible fungi has been widely reported, few studies have explored the mechanisms of selenium accumulation in *A. cornea* using NGS technology. In this study, the agronomic traits and nutrient concentrations in *A. cornea* treated with different amounts of selenium were investigated. The transcriptome of *A. cornea* at two typical stages (budding and mature) was explored to reveal the stage-specific mechanisms of selenium enrichment using NGS technology. Four differentially expressed genes that may affect the quality and agronomic traits of *A. cornea* were selected for RT-PCR validation, with the goal of providing a theoretical basis for future studies on selenium enrichment in edible fungi.

## Results

### Effect of selenium on the growth of *A. cornea*

**Figure 1:**
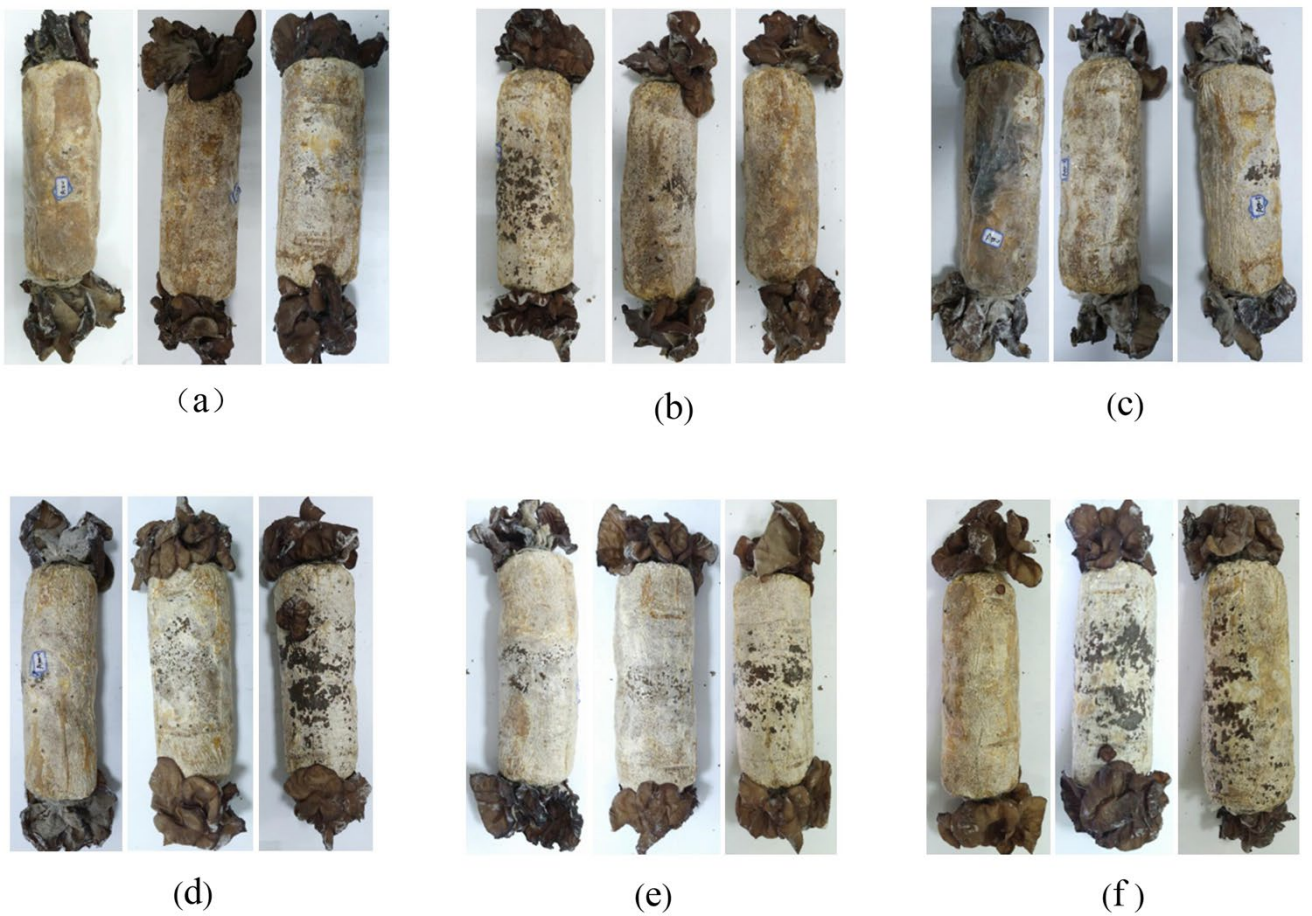
Fruiting bodies of *A. cornea* at the mature stage in different treatments. Abbreviations: (**a**) *ACK* the control group without selenium addition; (**b**) *A20* the treatment group with 20 µg/g sodium selenite addition; (**c**) *A50* the treatment group with 50 µg/g sodium selenite addition; (**d**) *A100* the treatment group with 100 µg/g sodium selenite addition; (**e**) *A150* the treatment group with 150 µg/g sodium selenite addition; (**f**) *A200* the treatment group with 200 µg/g sodium selenite addition.

Selenium addition affected the agronomic traits and nutrient contents of *A. cornea* (Table 1 and Fig. 1). The mean cluster weight increased first, then decreased following the increased concentration of selenium supply. It peaked (211.29 g) with the selenium concentration of 100 µg/g in the substrate, and was significantly higher than that of the control group. The mean size of a single random auricular patch was the biggest when supplied with 20 µg/g and 50 µg/g of selenium in the substrate. What was more, the mean fresh yield exhibited a first increase and afterward decrease trend, and it peaked with 100 µg/g of selenium augmented in the substrate, 47.05% higher than that of control group (473.99 g). However, the mycelial growth rate and thickness of *A. cornea* were significantly lower in the selenium-treated groups than those of control group. The crude polysaccharide content in *A. cornea* reached the highest (23.69%) with 150 µg/g of selenium supplied in the substrate. The total selenium concentration in the fruiting body increased with the increasing concentration of selenium, and it peaked (50.88 µg/g) with 200 µg/g of selenium added in the substrate, equivalent to 106 folds of control group.

**Table 1:** A*. cornea*’s agronomic traits and nutrient content of fruiting bodies in different treatments

NO.	MGR (mm/d)	Size (cm^2^)	Thickness (mm)	SW(g)	CW (g)	AC	Yield (g)	CP (%)	TSC (µg/g)
ACK	4.89 ± 0.52b	87.86 ± 24.47a	1.64 ± 0.19b	42.60 ± 11.60a	172.40 ± 18.06a	7.70 ± 1.64a	473.99 ± 20.71a	7.31 ± 0.34a	0.48 ± 0.02a
A20	4.19 ± 0.50a	127.24 ± 33.04bc	1.34 ± 0.23a	43.83 ± 15.02a	167.39 ± 38.45a	6.50 ± 1.27a	563.71 ± 23.19ab	12.26 ± 1.45b	0.59 ± 0.02a
A50	4.53 ± 0.59a	136.25 ± 47.66c	1.32 ± 0.15a	52.42 ± 21.65a	194.52 ± 50.66ab	6.50 ± 1.78a	599.61 ± 9.47bc	21.44 ± 0.01c	8.5 ± 0.23b
A100	4.23 ± 0.37a	121.32 ± 41.29abc	1.35 ± 0.13a	50.65 ± 15.17a	211.29 ± 50.82b	7.20 ± 0.92a	697.00 ± 49.64c	22.45 ± 0.22de	32.24 ± 1.22c
A150	4.46 ± 0.67a	113.59 ± 43.98abc	1.26 ± 0.13a	46.41 ± 13.23a	198.64 ± 28.33ab	7.80 ± 2.04a	605.88 ± 42.88bc	23.69 ± 0.24e	43.24 ± 1.01d
A200	4.32 ± 0.63a	89.82 ± 34.50ab	1.26 ± 0.16a	38.81 ± 11.50a	163.42 ± 27.31a	6.90 ± 1.10a	633.41 ± 28.82bc	18.66 ± 0.05d	50.88 ± 0.55e

Abbreviations: *NO*. cultivating formula; *MGR* mycelial growth rate; *Size* the size of a single random auricular patch; *Thickness* the thickness of a single random auricular patch; *SW* the weight of a single random auricular patch; *CW* the weight of a random cluster; *AC* the amount of a random cluster; *Yield* the fresh yield per bag; *CP* the content of the crude polysaccharide in the mature fruiting bodies; *TSC* the total selenium concentration in the mature fruiting bodies; *ACK* the control group without the addition of selenium; *A20* the treatment group with 20 µg/g sodium selenite addition; *A50* the treatment group with 50 µg/g sodium selenite addition; *A100* the treatment group with 100 µg/g sodium selenite addition; *A150* the treatment group with 150 µg/g sodium selenite addition; *A200* the treatment group with 200 µg/g sodium selenite addition. Data with different lower-case letters display significant differences (p-value < 0.05) by the LSD method using a one-way ANOVA. *MGR*, *Size*, *Thickness*, *SW*, *CW*, *AC* and *Yield* were replicated 8–10 times, while *CP* and *TSC* were replicated 3 times.

Generally, *A. cornea* cultured in the substrate with 100 µg/g of selenium outperformed other treatments in terms of improved agronomic traits and rich nutrients. Besides, the total selenium contents were significantly different in four growing periods between A100 and CK (P < 0.05) (Table S1). The highest total selenium content was tested at budding stage (136.46 µg/g), and subsequently dropped to 32.24 µg/g at mature stage. Thus, the treatment was subsequently selected for transcriptome analysis to explore the effect of selenium accumulation on the gene expression in *A. cornea* at the budding and mature stages.

### Sequencing of *A. cornea* Transcriptome and *De Novo* Assembly

High-throughput RNA sequencing was used to overview the gene expression profiles of *A. cornea* between the selenium-treated samples at the concentration of 100 µg/g (A100) and the control (ACK) at the budding and mature stages based on the Illumina NextSeq500 platform. After quality examination and filtering, an average of 3.09 × 10^7^ clean reads per sample were obtained (Table S2). The clean reads were assembled to produce 7.77 × 10^5^ contigs, 3.06 × 10^5^ transcripts and 2.56 × 10^5^ unigenes (Table S3). The average sizes of them were 251.34 bp, 520.54 bp and 452.75 bp, respectively. The length of the unigenes ranged from 200 bp to over 5,000 bp. Those ranging from 200 to 700 bp accounted for about 87% of the total number of the unigenes according to the sequence length distribution analysis (Table S4). In general, the RNA-Seq data were of high quality for subsequent transcriptome analysis.

### Functional Annotation of *A. cornea* Transcriptome

All the assembled unigenes from clean reads were aligned against the sequences from five frequently used databases for functional annotation, including NR, GO, KEGG, eggNOG and SwissProt (Fig. S1). The proportions of the unigenes matched to the databases were 43.34% (NR), 14.90% (GO), 2.06% (KEGG), 34.33% (eggNOG) and 36.34% (SwissProt), respectively, with a common percentage of 0.73% in all the five databases.

A total of 110783 unigenes from *A. cornea* transcriptome were  matched to the reference sequences of 782 other species in the NR database. In particular, 42% of the total unigenes were found to match to those of *A. delicata* TFB-10046 SS5 (Fig. S2). In addition, the unigenes with more than 1% of significant hits included *Batrachochytrium dendrobatidis* JAM81, *Rhizopus delemar* RA 99–880, *Arthrobotrys oligospora* ATCC 24927, etc. (Table S5). The unigenes with homologs found from other species accounted for 37.49%.

**Figure 2:**
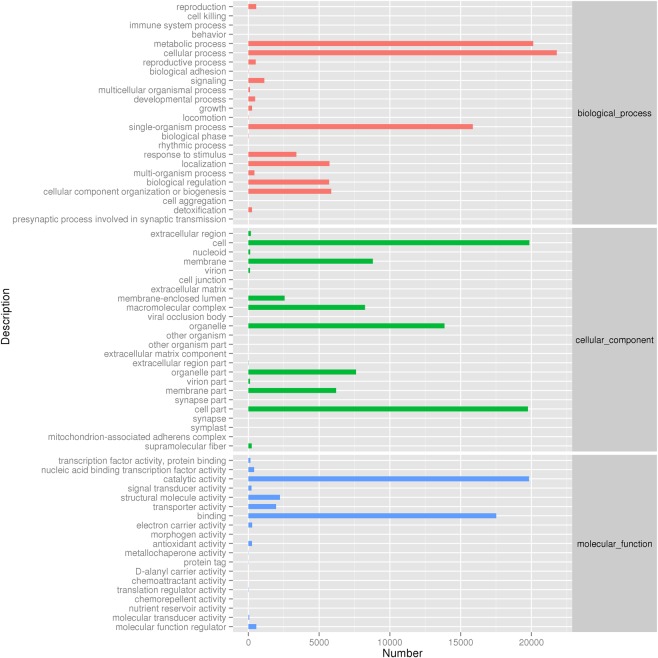
GO classification of the *A. cornea* transcriptome. X-axis represents the number of unigenes involved in the distinct GO terms. Y-axis displays the descriptions of GO terms.

A total of 213521 unigenes in the *A. cornea* transcriptome were annotated to GO database and grouped into 50 functional groups (Fig. 2). There were 19 functional groups involved in biological process, 16 in cellular component and 15 in molecular function, accounting for 38.54%, 41.08%, and 20.39% of the total annotated unigenes, respectively. Among all the GO classifications, “cellular process”, “metabolic process”, “cell”, “catalytic activity”, “cell part”, “binding”, “single-organism process”, “organelle”, “membrane” and “macromolecular complex” were high represented (Table S6). However, few genes related to “biological adhesion”, “locomotion”, “extracellular matrix”, “extracellular matrix component” and “nutrient reservoir activity” were found.

**Figure 3:**
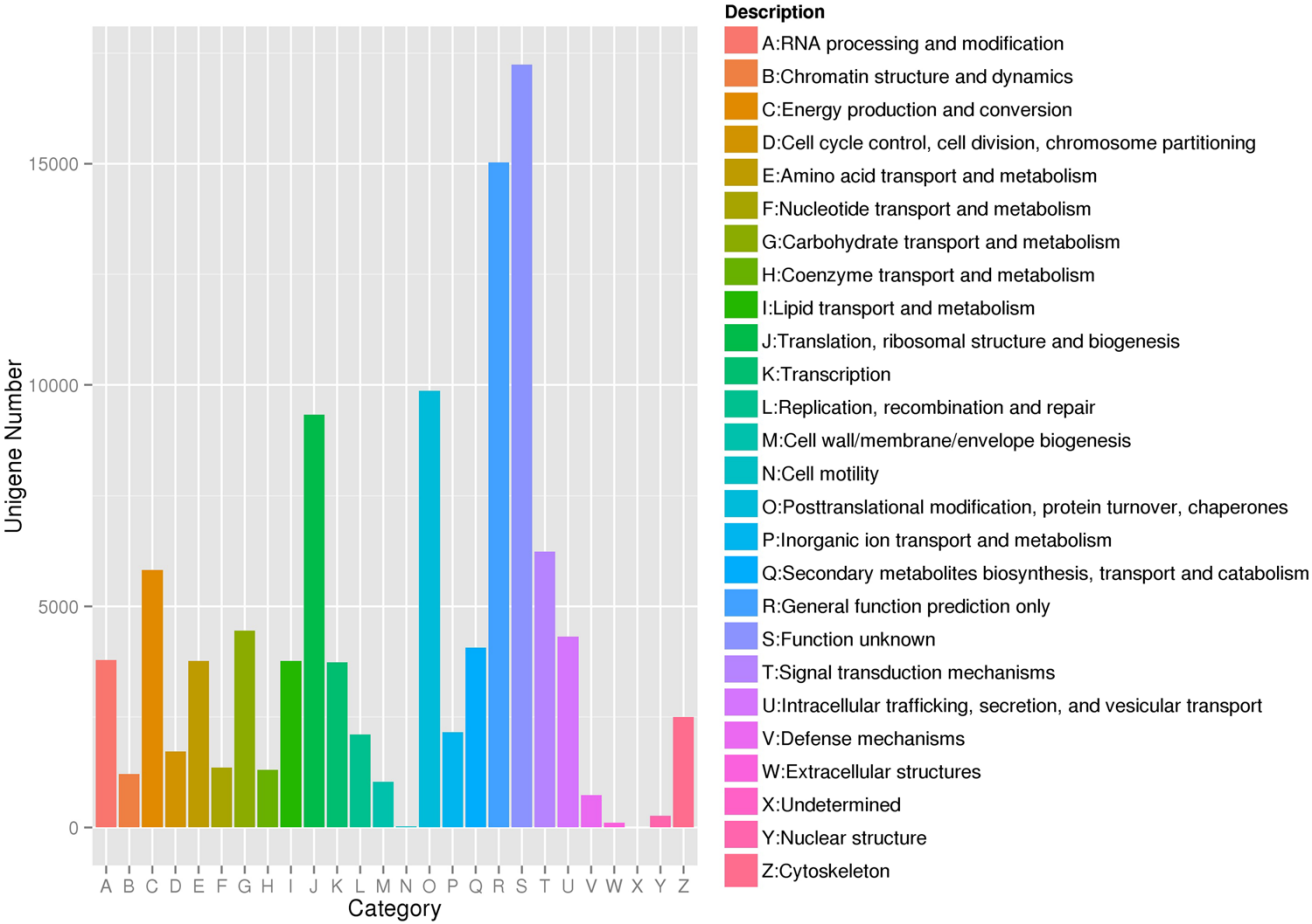
Classification of eggNOG annotations of the *A. cornea* transcriptome. The unigenes were annotated using eggNOG. The capital letters on the X-axis represent different eggNOG categories. Y-axis shows the number of unigenes in each eggNOG category.

To obtain more information on the functions of each protein, eggNOG analysis was performed. Finally, a total of 26 eggNOG categories was detected (Fig. 3). The categories of “function unknown” and “general function prediction only” were dominant, accounting for 16.28% and 14.18%, respectively.

**Figure 4:**
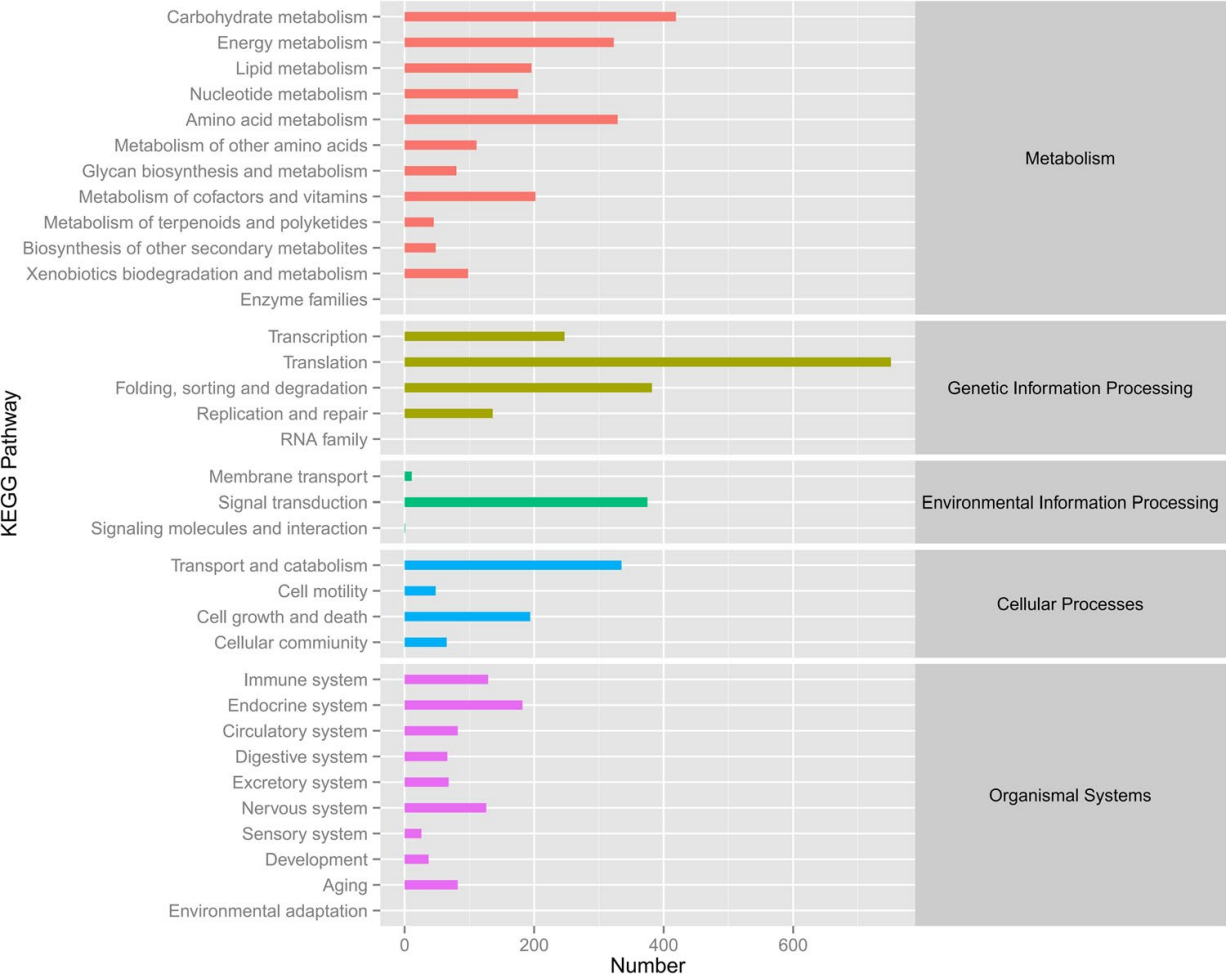
Classification of the KEGG pathways based on the *A. cornea* transcriptome. The grey blocks on the right represent the corresponding KEGG classes of the KEGG pathways.

The unigenes were mapped to the reference pathways in the KEGG database to identify the biological pathways in *A. cornea*. A total of 5721 unigenes were annotated and mapped to 35 KEGG pathways (Fig. 4 and Table S7). The pathways with the most unigenes in *A. cornea* were involved in “translation” (13.13%), “carbohydrate metabolism” (7.32%), “folding, sorting and degradation” (6.68%) and “signal transduction” (6.55%). None of the unigenes were assigned to the pathways of “enzyme families”, “RNA family” and “environmental adaptation”. The type of “metabolism” maintained the most unigenes, accounting for 41.57% in *A. cornea*.

The annotations to these databases provided a basic resource in *A. cornea*, and it aided further understanding of the effect of selenium accumulation on the biological activities in this fungus.

### Analysis of differentially expressed genes

**Figure 5:**
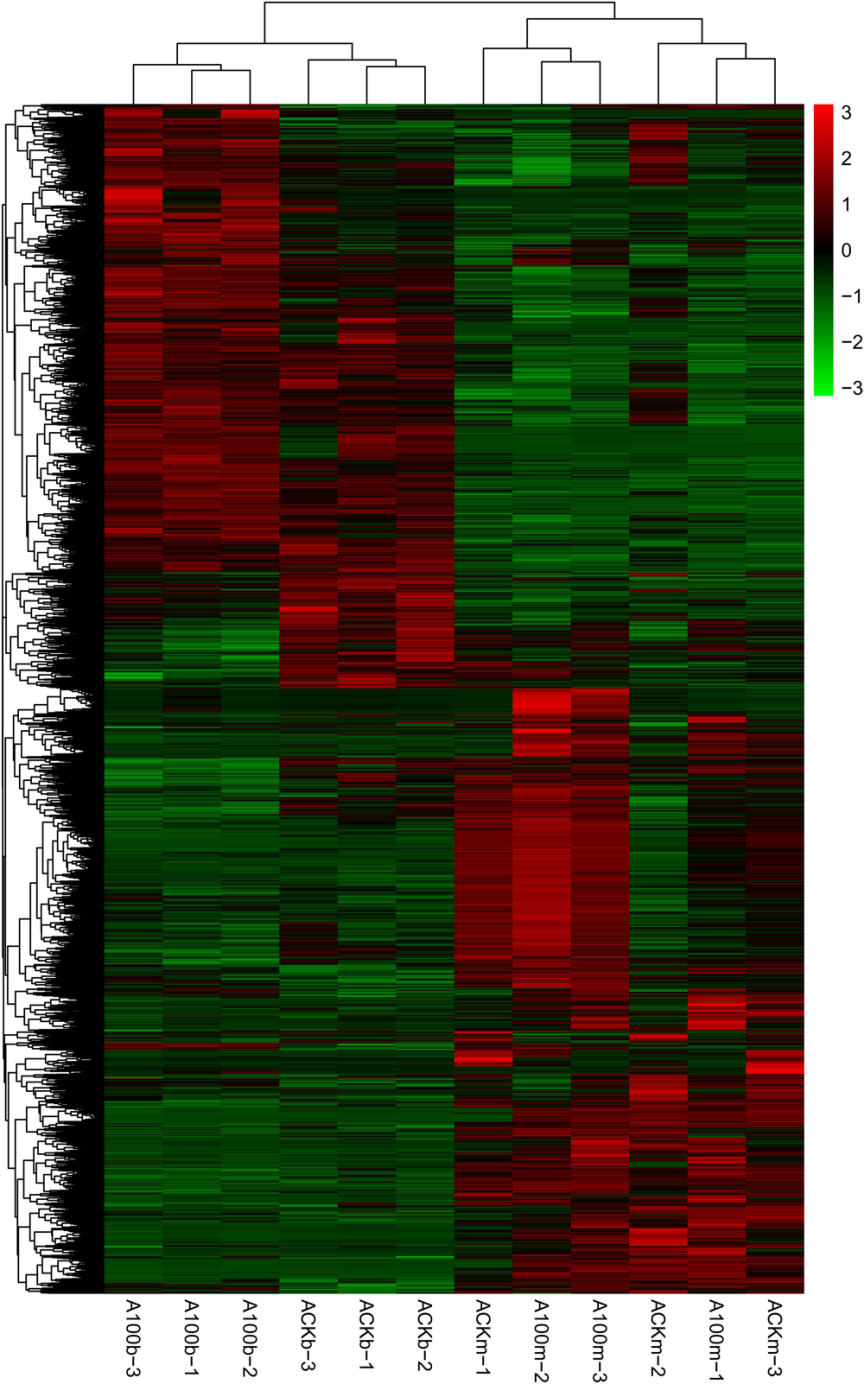
A heatmap showing the log_2_(FoldChange) values of the selenium-responsive DEGs (n = 3) in each sample. The DEGs and samples were subject to bidirectional clustering analysis using the R package Pheatmap based on the Euclidean distance and complete linkage clustering. The up-expressed DEGs are coloured in red and the down-regulated DEGs in green, respectively.

The differential gene expression patterns of *A. cornea* between the selenium-treated group (A100) and the control group (ACK) at the budding (b) and mature stages (m) were shown in Fig. 5. The RPKM method was applied to calculate the expression level of each unigene. The growth stage separated the *A. cornea* transcriptomes into two clusters. The one at the budding stage was further separated into two sub-clusters by two treatments. Interestingly, the up-expressed genes at the budding stage in both treatments became down-expressed at the mature stage. As illustrated in Fig. S3 and Table S8, selenium-related gene expression in *A. cornea* was more active at the budding stage than that at the mature stage. Among the 3907 differentially expressed unigenes (DEGs) between the selenium-treated and control groups at the budding stage, 1956 were up-expressed and 1951 were down-expressed. Besides, there were 891 selenium-responsive DEGs detected at the mature stage, including 575 up-expressed and 316 down-expressed unigenes.

**Figure 6:**
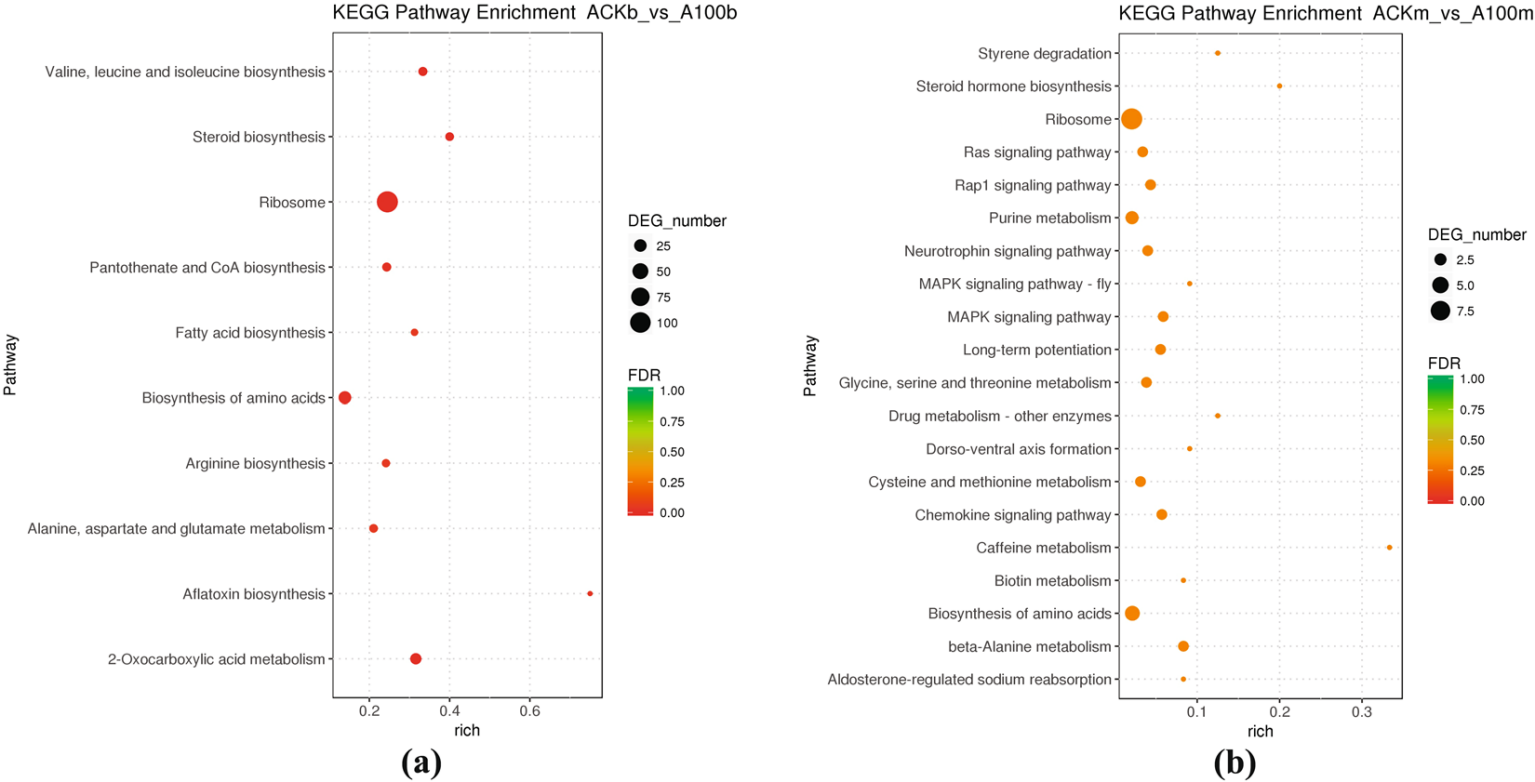
KEGG Pathway Enrichment of ACKb vs A100b (**a**) and ACKm vs A100m (**b**) *FDR*, false discovery rate adjusted p-value in multiple tests.

KEGG pathway enrichment analysis was used to illustrate the overrepresented metabolic and signalling pathways involved in the growth of *A. cornea* in response to the selenium supply at different growth stages (Table 2 and Fig. 6). At the budding stage, the majority of selenium-responsive DEGs involved in metabolism and genetic information processing pathways were up-expressed, representing 86.57% (116) and 98.13% (105), respectively. However, only a few selenium-responsive DEGs were related to metabolism (5) and genetic information processing (9) at the mature stage, of which, 4 pathways related to metabolism were up-expressed and 7 genetic information processing pathways were up-expressed. In addition, all the DEGs involved in the organismal systems (endocrine system) were up-expressed in the presence of selenium at the budding and mature stages (e.g., immune systems and nervous system).

**Table 2:** Overrepresented KEGG pathways between the selenium-treated and control groups of *A. cornea* at the budding and mature stages, respectively.

Pathway ID	KEGG name	Class Level 1	Class Level 2	Down-DEGs	Up-DEGs	Total DEGs	Total unigenes	p-value
***At the budding stage***								
ko04152	AMPK signaling pathway	Environmental Information Processing	Signal transduction	0	6	6	39	0.042
ko03010	Ribosome	Genetic Information Processing	Translation	2	105	107	437	0.000
ko00250	Alanine, aspartate and glutamate metabolism	Metabolism	Amino acid metabolism	0	8	8	38	0.003
ko00220	Arginine biosynthesis	Metabolism	Amino acid metabolism	2	5	7	29	0.002
ko00400	Phenylalanine, tyrosine and tryptophan biosynthesis	Metabolism	Amino acid metabolism	0	5	5	25	0.022
ko00350	Tyrosine metabolism	Metabolism	Amino acid metabolism	1	4	5	30	0.045
ko00290	Valine, leucine and isoleucine biosynthesis	Metabolism	Amino acid metabolism	1	8	9	27	0.000
ko00254	Aflatoxin biosynthesis	Metabolism	Biosynthesis of other secondary metabolites	0	3	3	4	0.001
ko00620	Pyruvate metabolism	Metabolism	Carbohydrate metabolism	0	10	10	62	0.007
ko00500	Starch and sucrose metabolism	Metabolism	Carbohydrate metabolism	6	4	10	75	0.025
ko00920	Sulfur metabolism	Metabolism	Energy metabolism	0	4	4	21	0.047
ko00061	Fatty acid biosynthesis	Metabolism	Lipid metabolism	0	5	5	16	0.003
ko00100	Steroid biosynthesis	Metabolism	Lipid metabolism	0	8	8	20	0.000
ko00770	Pantothenate and CoA biosynthesis	Metabolism	Metabolism of cofactors and vitamins	2	7	9	37	0.001
ko01210	2-Oxocarboxylic acid metabolism	Metabolism	Overview	3	15	18	57	0.000
ko01230	Biosynthesis of amino acids	Metabolism	Overview	3	23	26	187	0.000
ko01212	Fatty acid metabolism	Metabolism	Overview	0	7	7	51	0.050
ko04922	Glucagon signaling pathway	Organismal Systems	Endocrine system	0	6	6	40	0.046
***At the mature stage***								
ko04010	MAPK signaling pathway	Environmental Information Processing	Signal transduction	2	0	2	34	0.027
ko04015	Rap1 signaling pathway	Environmental Information Processing	Signal transduction	2	0	2	46	0.047
ko03010	Ribosome	Genetic Information Processing	Translation	7	2	9	437	0.005
ko04062	Chemokine signaling pathway	Organismal Systems	Immune system	2	0	2	35	0.029
ko04720	Long-term potentiation	Organismal Systems	Nervous system	2	0	2	36	0.030
ko00232	Caffeine metabolism	Metabolism	Biosynthesis of other secondary metabolites	1	0	1	3	0.023
ko00140	Steroid hormone biosynthesis	Metabolism	Lipid metabolism	1	0	1	5	0.037
ko00410	beta-Alanine metabolism	Metabolism	Metabolism of other amino acids	1	1	2	24	0.014

Abbreviations: *Down-DEGs* number of the down-expressed genes in response to selenium addition; *Up-DEGs* number of the up-expressed genes in response to selenium addition; *Total DEGs* total number of differentially expressed genes in response to selenium addition; *Total unigenes* total number of unigenes.

### Genes involved in metabolism

The metabolism-related pathways were overrepresented at the budding stage, and a large number of up-expressed selenium-related genes participated in these pathways. Among them, five were involved in amino acid metabolism. These associations included unigenes encoding for alanine, aspartate and glutamate metabolism (ko00250), arginine biosynthesis (ko00220), phenylalanine, tyrosine and tryptophan biosynthesis (ko00400), tyrosine metabolism (ko00350) and valine, leucine and isoleucine biosynthesis (ko00290). Besides, two pathways subordinated to lipid metabolism, including fatty acid biosynthesis (ko00061) and steroid biosynthesis (ko00100). Other metabolism-related pathways were also revealed up-expressed, like 2-Oxocarboxylic acid metabolism (ko01210), biosynthesis of amino acids (ko01230), pyruvate metabolism (ko00620), etc. However, the pathway of starch and sucrose metabolism (ko00500) showed down-expressed in response to the selenium supply. At the mature stage, three metabolism-related pathways displayed lower-expressed states, and they were caffeine metabolism (ko00232), steroid hormone biosynthesis (ko00140) and beta-alanine metabolism (ko00410).

### Genes involved in ribosome and other pathways

We found the greatest number of up-expressed unigenes in ribosome (ko03010) in response to the selenium supply at the budding stage. But the pathway of ribosome got lower-expressed at the mature stage. Other detected genes that significantly changed between treatments were mainly involved in enzyme activity and protein synthesis. At the budding stage, an average of 57.24% genes were related to protein synthesis (e.g. ribosomal protein, other predicted protein), and an average of 21.15% participated in enzyme activities (e.g. peroxidase, lipase, glycoside hydrolase), and a smaller portion of genes regulated other cellular life activities, such as transport, ATP, translation. It was noteworthy that a total of 7 genes that related to biological metabolism of glutathione exhibited up-expressed in the selenium-treated group.

### Validation of Transcriptome data by RT-qPCR

To validate the gene expression profiles in transcriptome of *A. cornea*, four DEGs were selected for real-time reverse transcription PCR (RT-qPCR). The genes *c108476_g4* related to “biosynthesis of amino acids” and *c102698_g1* related to “metabolism of xenobiotics by cytochrome P450”were up-expressed in control group. While the gene *c96467_g1* related to “bile secretion” was down-expressed in control group. Furthermore, *c135295_g1*, annotated neither in GO nor KEGG database, upregulated in control group at both stages, in which, it was with a higher expression level at the budding stage. All of the four selected genes were successfully amplified, and the expression patterns were highly consistent with the DEG results obtained from the transcriptome analysis, confirming the reliability of the DEG analysis from RNA-Seq data (Table 3).

**Table 3:** Unigenes used for validation of gene expression profiles in *A. cornea* transcriptome.

Group	Gene ID	DESeq analysis based on RNA-seq	Validation of the DEGs by qRT-PCR analysis			
		Log_2_Fold Change	p-value	ACK (2-*ΔΔ*Ct)	A100 (2-*ΔΔ*Ct)	p-value
A100b vs ACKb	c135295_g1	−1.65	1.33E-05	1.00 ± 0.11	0.72 ± 0.08	9.14E-03
	c108476_g4	−2.45	9.44E-10	1.00 ± 0.11	0.30 ± 0.05	5.89E-07
	c102698_g1	−3.07	8.07E-14	1.00 ± 0.02	0.14 ± 0.04	3.13E-08
	c96467_g1	2.25	1.78E-08	1.01 ± 0.13	9.76 ± 0.27	1.80E-27
A100m vs ACKm	c135295_g1	−6.12	8.45E-08	1.01 ± 0.13	0.01 ± 0.00	2.41E-09

2-*ΔΔ*Ct displays relative gene expression level using RPL4 as a reference gene in RT-qPCR analysis. Data are presented as the means ± standard deviation of three replicates. Abbreviations: *ACK* control group without selenium addition in the substrate; *A100* treatment group with 100 µg/g of selenium addition in the substrate; *m* at the mature stage; *b* at the budding stage.

## Discussion

### Selenium supply affects *A. cornea* physiological growth

In the present study, *A. cornea* maintained its normal growth with selenium supplementation in the substrate ranging from 20 µg/g to 200 µg/g. However, the mycelial growth of *A. cornea* slowed down in response to selenium supplementation. Malinowska *et al*. found that sodium selenite with low concentration of 25 mg/L did not significantly affect mycelial growth of *Hericium erinaceum*, whereas larger supplementation of Na_2_SeO_3_ resulted in a mycelial yield decrease^32^, which was consistent with our study. Furthermore, increased selenium supplementation caused thinning and extensive branching of mycelia of *G. lucidum*^33^. Besides, a dynamic change of selenium enrichment was observed, and selenium was finally accumulated efficiently in *A. cornea*. According to Goyal *et al*., the uptake of selenium in mycelia of *G. lucidum* showed an increasing trend as selenium supplementation increased^33^. The selenium absorption of mycelia might consequently affect selenium accumulation in fruiting bodies, and its accumulation differs from edible fungi. *G. lucidum* was reported to contain up to 72 μg Se/g in its fruiting body^34^, while the maximum selenium content in the studied *A. cornea* was approximately 51 μg Se/g. Moreover, the selenium concentration in *Lentinula edodes* first ascended and then descended over time^35^, which was consistent with the present study.

The present study revealed richer polysaccharides in fruiting bodies of *A. cornea* grown in the selenium-treated substrate. As a significant trace element, selenium is usually supplemented into the mushroom media, and the inorganic selenium is then bio-absorbed and subsequently transformed to organically bound one^36^. Selenium is demonstrated to bind with proteins and polysaccharides^37^, or form a conjugated complex with polysaccharides of edible fungi^38^. Chen *et al*. reported that, sodium selenite supplementation in the media of *Spirulina platensis* contributed an increase to extracellular polysaccharides in dose-dependent manner, and the extracellular polysaccharide was probably produced by mycelia in response to oxidative damage^39^. Additionally, the synthesis of proteins and polysaccharides in *G. lucidum* was enhanced with the selenium concentration below 100 ug/g in the substrate^15^. Consequently, a proper range of selenium supplementation is suggested for *A. cornea* cultivation to improve the agronomic properties (e.g. fresh yield, polysaccharide content).

### *De Novo* assembly of transcriptome reveals the close relationship between *A. cornea* and *A. delicate*

The GC content of *A. cornea* was approximately 57.93%, which was lower than what was reported by Zhou *et al*. (61.3%)^40^. No reference genome was found, so an approximation of transcriptome coverage for assembly was made. Based on NR database, most hit genes were obtained in *A. delicata* TFB-10046 SS5. The two species were highly similar in genes, which was consistent with Zhou *et al*. study^40^. Furthermore, none of the four tested restriction enzymes including Hae ill, Taq i, Hinfl and Msp 1.ms could separate *A. cornea* and *A. delicate*, suggesting their exact relationship of affinis species^41^. Besides, the unigenes with more than 1% of significant hits included *Batrachochytrium dendrobatidis* JAM81, a fungus that affects the occurrence of Amphibians; *Rhizopus delemar* RA 99–880, a fungus that belongs to the Zygomycota; *Arthrobotrys oligospora* ATCC 24927; *Dichomitus squalens* LYAD-421 SS1 and three *Trichoderma* species.

### Functional annotation reveals the importance of protein synthesis throughout the growth of *A. cornea*

The three databases GO, GOSlim and KEGG complemented for functional annotation of genes. GO analysis presented catalytic activity as one important functional category during the growth of *A. cornea*. Various enzymes, including cellulose, lignin peroxidase, were involved in catalysis during mushroom growth, and catalytic activity dominated such biological processes like metabolism, nutrition and energy conversion^42–44^. The present study revealed the largest number of unigenes involved in translation, agreeing with the previous study^40^. Translation is considered as an indispensable process in the genetic central dogma and plays a key role in biological growth. Notably, two KEGG pathways “ribosome” and “biosynthesis of amino acids” were detected at both budding and mature stages, suggesting the significance of protein synthesis during these two periods.

### Expression of the genes is growth stage dependent

Interestingly, we found a higher number of DEGs at the budding stage than at the mature stage. And the up-expressed genes at the budding stage had lower expression level at the mature stage. Different gene expression patterns indicated their significance at the respective developmental stages of mushrooms. Some genes function differently at different growth stages^45,46^. Based on the transcriptional analysis of *G. lucidum*, numerous metabolic pathways involved in biosynthesis upregulated during the mycelium stage, whereas, unigenes related to degradation activity were over-expressed during the fruiting body stage^27^. Morin *et al*. compared gene expression in *Agaricus bisporus* of fruiting bodies and undifferentiated mycelium, and found genes coding for hydrophobins, lectins were over-expressed at fruiting body stage^47^. Besides, the transcriptomes of *Hypsizygus marmoreus* at four growth stages revealed that, stress signals including MAPK, cAMP might be the most important pathways during the fruiting body stage^26^. What’s more, in analysis of DEGs in *Agrocybe aegerita* transcriptome, Wang *et al*. uncovered genes related to polysaccharide and steroid biosynthesis were differentially expressed at mycelium and fruiting body stages^48^. In the present study on *A. cornea*, unigenes involved in biosynthesis of amino acids were high expressed at the budding stage, and the ribosome pathway relating to translation was in high expression at the budding and mature stages.

Physiological metabolisms vary in different growth periods, and all kinds of genes are responsible to regulate and control diverse life activities^49,50^. At the budding stage of *A. cornea*, reproductive growth dominates with primordium differentiation. Instead, nutrients are enriched in fruiting bodies at the mature stage. As one of the most complex and rapid developmental stage of mushroom growth, the fruiting process is reported to be regulated by cellular processes^51^. At this growth stage, dramatic morphological changes are caused by complicated regulations on transcription and translation^52^. And fruiting body development is often motivated by environmental changes^53^. It’s inferred that, most of physiological activities in *A. cornea* tend to slow down or even cease when its tissues get mature at the mature stage. As a result, massive unigenes are down-expressed or shutdown.

### Selenium supply significantly affects the metabolism of *A. cornea*

The transcriptome analysis was performed to show the effect of selenium addition on *A.cornea* at different growth stages. Selenium stimulated the synthesis of various proteins, and it would improve the medical use of *A. cornea*. Selenium was absorbed and converted into various substances, including selenocysteine, selenomethionine and other compounds^35^. It was demonstrated that the accumulated selenium in *G. lucidum* protein showed antioxidant activities and selenium protected DNA from being oxidized strongly^54^. In the present study, 7 genes related to biological metabolism of glutathione were up-expressed. As was reported, selenium contributes to be part of selenoproteins, especailly glutathione peroxidase^55^. And glutathione peroxidase contributes to protect exposed organisms from damage caused by reactive oxygen species^56^. In addition, selenium upregulated genes of glucanase synthesis in the present study. Accordingly, selenium promoted adenosine accumulation in *Cordyceps militaris* during its growth^57^. Meanwhile, one of the selected genes (*c102698_g1*) for verification was related to “metabolism of xenobiotics by cytochrome P450”. Cytochrome P450 (CYPs) is considered as an important role for biological metabolism. Morin *et al*. detected genes of cytochrome P450 in *Agaricus bisporus* grown on compost, with a relatively low number but in high expression^58^.

### The significance of the present study

Edible mushrooms are good for human health and longevity due to their natural antioxidant compounds^59,60^. *A. cornea* is a particularly popular edible mushroom, and has attracted substantial interest^21,61^. Most of the edible fungi have a strong ability to enrich mineral elements^62,63^. The enriched elements have been demonstrated to increase the nutritive value of edible fungi, among which selenium is an outstanding one^64,65^. As an essential trace element for humans, selenium is feasible to be added into the substrates for producing selenium-biofortified mushrooms with great agronomic value^66^. Mushrooms including *Agaricus bisporus*, *Lentinula edodes*, are capable of accumulating selenium through enzymatic digestion and transforming it into organic components that are beneficial to human^67–70^. The bioavailability of organically bound selenium from dietary supplements is better with lower toxicity for the mushrooms than inorganic selenium compounds^5^. The effect of selenium on *A. cornea* in the present study strongly supported the above-mentioned views. The quality of *A. cornea* in the selenium-treated cultivation substrate is strongly enhanced by the accumulation of this trace element and indirectly by the selenium-enhanced biological activities. The present study provides a solid support for inorganic selenium addition into the substrate during the commercial cultivation of *A. cornea* in agricultural practice.

## Methods

### Cultivation of *A. cornea*

The *A. cornea* cultivar named Shanghai No. 1 was provided by the Soil and Fertilizer Research Institute, Sichuan Academy of Agricultural Science. The substrate comprised cottonseed hull (10%), corn cob (30%), sawdust (33%), rice bran (20%), corn flour (2%), gypsum (1%) and lime (4%), and all the materials were fresh, dry and unspoiled. A sodium selenite (Na_2_SeO_3_) solution was added to the substrate as the selenium source. After thorough mixing, the final concentrations of sodium selenite in the substrate were determined to be 20 µg/g, 50 µg/g, 100 µg/g, 150 µg/g, and 200 µg/g and were labelled A20, A50, A100, A150, A200, respectively. The control group without the addition of selenium to the substrate was labelled ACK. The substrate was placed in polyethylene cultivation bags (size: 42 cm × 22 cm × 0.005 cm) and atmospheric sterilized at 100 °C for 14 hours. After sterilization, the bags were cooled to room temperature and placed in a laminar flow hood for the inoculation of *A. cornea*. After inoculation, they were planted in the cultivation base of Jiandi, Shifang, China (N 31°12′20.33″, E 104°01′42.35″). The cultivation area was ventilated. The cultivation area had been cleaned and disinfected before the experiment was conducted. The mycelial growth rate of *A. cornea* was measured at the hyphal stage. Other agronomic traits including the size, thickness and weight of a single random auricular patch, fresh yield, crude polysaccharide and total selenium concentration in the fruiting bodies in the different treatments were investigated at the mature stage. There were three replicates per treatment. We used 15 cultivation bags in each replicate and a total of 270 cultivation bags were used for the study. Excel and SPSS13.0 were used for statistical analysis. Data with different lower-case letters display significant differences (p-value < 0.05) by the LSD method of a one-way ANOVA.

### Sample collection for transcriptome analysis

The tissues of *A. cornea* cultured in ACK and A100 were sampled with three replicates at the budding and mature phases and denominated ACKb, ACKm, A100b and A100m. Disposable disinfected gloves, sterilized tweezers and knives were prepared and used to take samples. Fresh tissues of the same treatment were collected and pooled, weighing over 500 mg per sample. The samples were filled in 2 mL Eppendorf tubes (Eppendorf, Germany), stored in liquid nitrogen and immediately sent to Personalbio (Shanghai, China) for RNA extraction and transcriptome sequencing.

### RNA extraction, library preparation and sequencing

Total RNA from the tissues of *A. cornea* was extracted using a Qiagen RNeasy mini kit (Qiagen, Germany) including DNAase treatment following the manufacturer’s instructions. The quality of the extracted RNA was examined using agarose gel electrophoresis. The ribosomal RNA that had contaminated the total RNA was eliminated using a Ribosomal RNA Removal Kit (Illumina, San Diego, CA, USA). The remaining RNA was cleaved into small fragments with lengths of 200–300 bp. The RNA was reverse transcribed to cDNA using a Fast Quant RT Kit (with gDNAase) (TIANGEN, Beijing) according to the manufacturer’s instructions. The first strand of cDNA was synthesized with random hexamers and reverse transcriptase using RNA as the template. The second strand was synthesized based on the first strand with the base T replaced by U. To obtain more accurate results for the subsequent analysis in the gene functional annotation and expression, a TruSeq Stranded mRNA Library Prep Kit (Illumina, San Diego, CA, USA) that can determine the direction of justice chains and antisense chains was adopted to construct the libraries^71^

The cDNA fragments were enriched using PCR amplification after the generation of the library. The libraries with fragment lengths between 300 and 400 bp were examined using an Agilent 2100 Bioanalyzer (Agilent Technologies, CA, USA). The total and effective concentrations of the libraries were determined. The libraries with varied index sequences were pooled in proportion based on the effective concentrations of the libraries and the amount of data required by the libraries. The pooled libraries were uniformly diluted to 2 nM and formed single stranded libraries using alkaline denaturation. Finally, the libraries were paired-end sequenced based using an Illumina NextSeq500 (Illumina, San Diego, CA, USA). All the raw sequences were deposited in the NCBI Sequence Read Archive (SRA) database with the accession NO. SAMN07184775-SAMN07184786.

### *De novo* transcriptome assembly and gene functional annotation

Raw reads in the FASTQ files were checked and filtered by quality control using the FastQC program^72^ The adapters were removed from the sequences. The sequences with a length less than 50 bp and with an average sequence quality score lower than Q20 were removed. Trinity^73^, a short reads assembling program, was used to perform *de novo* assembly of the clean reads as described by Zhou^40^. High-quality sequences were alternatively spliced from the beginning to obtain transcript sequences using Inchworm, Chrysalis and Butterfly. The longest sequence of every cluster was selected as one unigene, which was finally annotated against the databases of GO (Gene Ontology)^74^, KEGG (Kyoto Encyclopedia of Genes and Genome)^75^, eggNOG (evolutionary genealogy of genes: Non-supervised Orthologous Groups)^76^ and SwissProt (Swiss-Prot protein)^77^. The alignment result (the read count of each unigene) was obtained by aligning the clean sequences with the corresponding unigene for the following differential gene expression and enrichment analysis.

### Differentially expressed genes

The expression quantification of RNA-Seq data was performed using RSEM, software for accurate transcript quantification^78^. The *de novo* assembled transcriptome was used as a reference. The alignment result was investigated. The FPKM value of each unigene was calculated as an estimation of the expression level. Within RNA-Seq technology, FPKM represents the expected number of fragments per kilobase of transcript sequence per million reads sequenced^79^. The differences in unigene expression were analysed using DESeq (Version 1.18.0)^78^. The significantly differentially expressed genes (DEGs) were screened with the threshold: |log_2_(FoldChange)| > 1 and p-value < 0.05. Two-way hierarchical clustering (complete linkage) was conducted to show the expression pattern of each DEG across all the samples using the R package Pheatmap^80^ based on the Euclidean distance. Volcano plots were drawn to show the patterns of unigene expression between the selenium-treated (A100) and control groups (ACK) at the budding and mature stages using the R package ggplot2^81^.

### Functional enrichment analysis

GO term enrichment analysis was conducted using the GOseq R packages based on Wallenius non-central hyper-geometric distribution^82^. All the DEGs were first assigned to a set of predefined Terms of Gene Ontology (TOG) to profile their biological functions. The TOGs overrepresented with DEGs were examined compared to a randomly sampled set of genes using hypergeometric testing^83^. The KEGG pathways enrichment analysis was performed similarly based on the hypergeometric distribution principle using KOBAS software^84^. A p-value < 0.05 was used as the threshold to determine the significance of the enrichment pattern of each TOG or KEGG pathway between the selenium treated (A100) and control groups (ACK) at the budding and mature stages, respectively.

### Validation of differentially expressed genes by RT-qPCR

A total of four unigenes identified by RNA-Seq were selected for expression validation using the RT-qPCR analysis. Approximately 3 mg of RNA of each sample was reversely transcribed using a Super RT Kit (TaKaRa, Osaka, Japan), following the manufacturer’s instructions. The sequences of the primers used to amply the selected genes are shown in Table S9. RPL4 (Ribosomal Protein L4) was amplified as a reference gene to normalize gene expression for each RT-PCR using primers described by Xu J *et al*.^85^. At least three independent RT-PCR reactions were performed to confirm the expression of each gene in one sample.

## Supplementary information

Supplementary Figure 1

Supplementary Figure 2

Supplementary Figure 3

Supplementary Table 1

Supplementary Table 2

Supplementary Table 3

Supplementary Table 4

Supplementary Table 5

Supplementary Table 6

Supplementary Table 7

Supplementary Table 8

Supplementary Table 9
