# Supplementary material for: Transcriptional profiling of Auricularia cornea in selenium accumulation
Source: Sci Rep. 2019 Apr 4;9:5641. doi: 10.1038/s41598-019-42157-2 (PMC6449350; doi:10.1038/s41598-019-42157-2)
Supplement: Supplementary file 3 — Supplementary Figure 3 [file 41598_2019_42157_MOESM3_ESM.pdf]

Transcriptional profiling of *Auricularia cornea* in selenium accumulation

Xiaolin Li[1#*], Lijuan Yan[2#], Qiang Li[3,4], Hao Tan[1], Jie Zhou[1], Renyun Miao[1], Lei Ye[1], Weihong Peng[1], Xiaoping Zhang[5], Wei Tan[1*], Bo Zhang[1*]

[1] Soil and Fertilizer Institute, Sichuan Academy of Agriculture Sciences, Chengdu 610066, China;

[2] Chair for Aquatic Geomicrobiology, Institute of Biodiversity, Friedrich Schiller University Jena, Jena, D-07743, Germany

[3] Biotechnology and Nuclear Technology Research Institute, Sichuan Academy of Agricultural Sciences, Chengdu 610061, China

[4] College of Life Science, Sichuan University, Chengdu 610065, China

[5] Department of Microbiology, College of Resources, Sichuan Agricultural University, Chengdu 611130, China;

[#] Xiaolin Li and Lijuan Yan contributed equally to the work.

* correspondence: Xiaolin Li kerrylee_tw@sina.com

Wei Tan tanweichengdu@126.com

Bo Zhang bozhang5658@foxmail.com

Table S3 Length distribution of contigs, transcripts and unigenes of *Auricularia cornea*

	**Contig**	**Transcript**	**Unigene**
Total Length (bp)	1.95×10^{8}	1.59×10^{8}	1.16×10^{8}
Sequence Number	7.77×10^{5}	3.06×10^{5}	2.56×10^{5}
Max. Length (bp)	11959	15115	15115
Mean Length (bp)	251.34	520.54	452.75
N50 (bp)	256	685	501
N50 Sequence No.	162,87	58,44	54,25
N90 (bp)	132	242	233
N90 Sequence No.	6.06×10^{5}	2.33×10^{5}	2.02×10^{5}
GC%	57.43	58.74	57.93
