# Supplementary material for: Transcriptional profiling of Auricularia cornea in selenium accumulation
Source: Sci Rep. 2019 Apr 4;9:5641. doi: 10.1038/s41598-019-42157-2 (PMC6449350; doi:10.1038/s41598-019-42157-2)
Supplement: Supplementary file 4 — Supplementary Table 1 [file 41598_2019_42157_MOESM4_ESM.pdf]

Transcriptional profiling of *Auricularia cornea* in selenium accumulation

Xiaolin Li[1#*], Lijuan Yan[2#], Qiang Li[3,4], Hao Tan[1], Jie Zhou[1], Renyun Miao[1], Lei Ye[1], Weihong Peng[1], Xiaoping Zhang[5], Wei Tan[1*], Bo Zhang[1*]

[1] Soil and Fertilizer Institute, Sichuan Academy of Agriculture Sciences, Chengdu 610066, China;

[2] Chair for Aquatic Geomicrobiology, Institute of Biodiversity, Friedrich Schiller University Jena, Jena, D-07743, Germany

[3] Biotechnology and Nuclear Technology Research Institute, Sichuan Academy of Agricultural Sciences, Chengdu 610061, China

[4] College of Life Science, Sichuan University, Chengdu 610065, China

[5] Department of Microbiology, College of Resources, Sichuan Agricultural University, Chengdu 611130, China;

[#] Xiaolin Li and Lijuan Yan contributed equally to the work.

* correspondence: Xiaolin Li kerrylee_tw@sina.com

Wei Tan tanweichengdu@126.com

Bo Zhang bozhang5658@foxmail.com

Table S1 Total selenium content in *Auricularia cornea* at four growing stages

NO.	Total selenium content at different stages (μg/g)			
	Hypha stage	Budding stage	Spreading stage	Mature stage
ACK	0.47±0.01b	0.36±0.01a	0.75±0.01c	0.48±0.01b
A100	68.15±1.37b	136.46±0.98d	87.51±0.78c	32.24±0.70a

Data with different lower-case letters display significant differences (p-value < 0.05) by the LSD method using a one-way ANOVA. Total selenium content at different stages replicated 3 times. Abbreviations: *ACK* control group without selenium addition in the substrate; *A100* treatment group with 100 μg/g of selenium addition in the substrate.
