# Supplementary material for: Transcriptional profiling of Auricularia cornea in selenium accumulation
Source: Sci Rep. 2019 Apr 4;9:5641. doi: 10.1038/s41598-019-42157-2 (PMC6449350; doi:10.1038/s41598-019-42157-2)
Supplement: Supplementary file 5 — Supplementary Table 2 [file 41598_2019_42157_MOESM5_ESM.pdf]

Transcriptional profiling of *Auricularia cornea* in selenium accumulation

Xiaolin Li[1#*], Lijuan Yan[2#], Qiang Li[3,4], Hao Tan[1], Jie Zhou[1], Renyun Miao[1], Lei Ye[1], Weihong Peng[1], Xiaoping Zhang[5], Wei Tan[1*], Bo Zhang[1*]

[1] Soil and Fertilizer Institute, Sichuan Academy of Agriculture Sciences, Chengdu 610066, China;

[2] Chair for Aquatic Geomicrobiology, Institute of Biodiversity, Friedrich Schiller University Jena, Jena, D-07743, Germany

[3] Biotechnology and Nuclear Technology Research Institute, Sichuan Academy of Agricultural Sciences, Chengdu 610061, China

[4] College of Life Science, Sichuan University, Chengdu 610065, China

[5] Department of Microbiology, College of Resources, Sichuan Agricultural University, Chengdu 611130, China;

[#] Xiaolin Li and Lijuan Yan contributed equally to the work.

* correspondence: Xiaolin Li kerrylee_tw@sina.com

Wei Tan tanweichengdu@126.com

Bo Zhang bozhang5658@foxmail.com

Table S2 Throughput and quality of Illumina sequencing of *Auricularia cornea* transcriptome

Sample	Raw Reads	**Clean Reads**	Raw Bases (bp)	**Clean** Bases **(bp)**	N (%)	Q20 (%)	Q30 (%)	GC (%)
ACKb-1	$2.71*10^7$	$2.70*10^7$	$4.07*10^9$	$3.89*10^9$	$2.35*10^{-3}$	97.92	94.55	60.00
ACKb-2	$3.38*10^7$	$3.36*10^7$	$5.07*10^9$	$4.79*10^9$	$2.31*10^{-3}$	97.84	94.38	61.86
ACKb-3	$2.79*10^7$	$2.78*10^7$	$4.19*10^9$	$4.10*10^9$	$1.25*10^{-3}$	98.76	96.53	63.38
A100b-1	$3.13*10^7$	$3.11*10^7$	$4.69*10^9$	$4.39*10^9$	$2.39*10^{-3}$	97.97	94.72	62.24
A100b-2	$3.49*10^7$	$3.46*10^7$	$5.23*10^9$	$4.93*10^9$	$2.51*10^{-3}$	97.87	94.45	62.14
A100b-3	$3.40*10^7$	$3.38*10^7$	$5.10*10^9$	$4.80*10^9$	$2.38*10^{-3}$	97.94	94.59	62.16
ACKm-1	$2.89*10^7$	$2.87*10^7$	$4.33*10^9$	$4.18*10^9$	$1.34*10^{-3}$	98.32	95.51	63.18
ACKm-2	$3.15*10^7$	$3.13*10^7$	$4.73*10^9$	$4.55*10^9$	$0.71*10^{-3}$	98.65	96.45	63.36
ACKm-3	$3.38*10^7$	$3.35*10^7$	$5.06*10^9$	$4.90*10^9$	$0.65*10^{-3}$	98.52	96.24	62.65
A100m-1	$2.62*10^7$	$2.61*10^7$	$3.93*10^9$	$3.84*10^9$	$3.60*10^{-3}$	98.49	96.15	62.80
A100m-2	$3.45*10^7$	$3.43*10^7$	$5.17*10^9$	$4.97*10^9$	$0.58*10^{-3}$	98.72	96.57	63.35
A100m-3	$2.81*10^7$	$2.79*10^7$	$4.21*10^9$	$4.04*10^9$	$0.61*10^{-3}$	98.79	96.75	63.23

Abbreviations: *ACK* control group without selenium addition in the substrate; *A100* treatment group with 100 µg/g of selenium addition in the substrate; *m* at the mature stage; *b* at the budding stage; *N*, the percentage of unclear bases, *Q20* the percentage of bases above 99% accuracy; the percentage of bases above 99.9% accuracy.
