# Supplementary material for: Transcriptional profiling of Auricularia cornea in selenium accumulation
Source: Sci Rep. 2019 Apr 4;9:5641. doi: 10.1038/s41598-019-42157-2 (PMC6449350; doi:10.1038/s41598-019-42157-2)
Supplement: Supplementary file 7 — Supplementary Table 4 [file 41598_2019_42157_MOESM7_ESM.pdf]

# Transcriptional profiling of *Auricularia cornea* in selenium accumulation

Xiaolin Li[1#*], Lijuan Yan[2#], Qiang Li[3,4], Hao Tan[1], Jie Zhou[1], Renyun Miao[1], Lei Ye[1], Weihong Peng[1], Xiaoping Zhang[5], Wei Tan[1*], Bo Zhang[1*]

[1] Soil and Fertilizer Institute, Sichuan Academy of Agriculture Sciences, Chengdu 610066, China;

[2] Chair for Aquatic Geomicrobiology, Institute of Biodiversity, Friedrich Schiller University Jena, Jena, D-07743, Germany

[3] Biotechnology and Nuclear Technology Research Institute, Sichuan Academy of Agricultural Sciences, Chengdu 610061, China

[4] College of Life Science, Sichuan University, Chengdu 610065, China

[5] Department of Microbiology, College of Resources, Sichuan Agricultural University, Chengdu 611130, China;

[#] Xiaolin Li and Lijuan Yan contributed equally to the work.

* correspondence: Xiaolin Li kerrylee_tw@sina.com

Wei Tan tanweichengdu@126.com

Bo Zhang bozhang5658@foxmail.com

**Table S4 Investigation of length distribution for every 100 bp of contigs, transcripts and unigenes**

| Length (bp) | Contig | Transcript | Unigene | Length (bp) | Contig | Transcript | Unigene | Length (bp) | Contig | Transcript | Unigene |
|---|---|---|---|---|---|---|---|---|---|---|---|
| 100:199 | 517745 | 0 | 0 | 1800:1899 | 893 | 1651 | 948 | 3500:3599 | 151 | 144 | 81 |
| 200:299 | 142021 | 142643 | 133928 | 1900:1999 | 776 | 1396 | 786 | 3600:3699 | 135 | 105 | 60 |
| 300:399 | 45831 | 51544 | 45569 | 2000:2099 | 645 | 1126 | 641 | 3700:3799 | 93 | 96 | 58 |
| 400:499 | 21071 | 26549 | 21676 | 2100:2199 | 612 | 953 | 555 | 3800:3899 | 124 | 85 | 48 |
| 500:599 | 11495 | 16284 | 12359 | 2200:2299 | 498 | 792 | 432 | 3900:3999 | 102 | 59 | 43 |
| 600:699 | 7328 | 11469 | 8119 | 2300:2399 | 417 | 635 | 355 | 4000:4099 | 85 | 59 | 39 |
| 700:799 | 4999 | 8683 | 5809 | 2400:2499 | 403 | 578 | 306 | 4100:4199 | 80 | 57 | 34 |
| 800:899 | 3524 | 6818 | 4285 | 2500:2599 | 368 | 526 | 290 | 4200:4299 | 68 | 44 | 32 |
| 900:999 | 2884 | 5507 | 3339 | 2600:2699 | 302 | 441 | 226 | 4300:4399 | 71 | 50 | 34 |
| 1000:1099 | 2304 | 4738 | 2772 | 2700:2799 | 291 | 396 | 237 | 4400:4499 | 60 | 50 | 25 |
| 1100:1199 | 1960 | 4228 | 2455 | 2800:2899 | 237 | 275 | 152 | 4500:4599 | 48 | 28 | 18 |
| 1200:1299 | 1665 | 3554 | 2003 | 2900:2999 | 265 | 302 | 168 | 4600:4699 | 57 | 41 | 23 |
| 1300:1399 | 1488 | 3140 | 1758 | 3000:3099 | 181 | 239 | 146 | 4700:4799 | 53 | 16 | 15 |
| 1400:1499 | 1383 | 2655 | 1516 | 3100:3199 | 214 | 183 | 105 | 4800:4899 | 42 | 15 | 9 |
| 1500:1599 | 1267 | 2361 | 1314 | 3200:3299 | 160 | 185 | 108 | 4900:4999 | 32 | 9 | 3 |
| 1600:1699 | 1145 | 2258 | 1247 | 3300:3399 | 162 | 162 | 101 | >5000 | 401 | 185 | 115 |
| 1700:1799 | 1058 | 2030 | 1142 | 3400:3499 | 157 | 172 | 106 | | | | |
