# Supplementary material for: Transcriptional profiling of Auricularia cornea in selenium accumulation
Source: Sci Rep. 2019 Apr 4;9:5641. doi: 10.1038/s41598-019-42157-2 (PMC6449350; doi:10.1038/s41598-019-42157-2)
Supplement: Supplementary file 8 — Supplementary Table 5 [file 41598_2019_42157_MOESM8_ESM.pdf]

Transcriptional profiling of *Auricularia cornea* in selenium accumulation

Xiaolin Li[1#*], Lijuan Yan[2#], Qiang Li[3,4], Hao Tan[1], Jie Zhou[1], Renyun Miao[1], Lei Ye[1], Weihong Peng[1], Xiaoping Zhang[5], Wei Tan[1*], Bo Zhang[1*]

[1] Soil and Fertilizer Institute, Sichuan Academy of Agriculture Sciences, Chengdu 610066, China;

[2] Chair for Aquatic Geomicrobiology, Institute of Biodiversity, Friedrich Schiller University Jena, Jena, D-07743, Germany

[3] Biotechnology and Nuclear Technology Research Institute, Sichuan Academy of Agricultural Sciences, Chengdu 610061, China

[4] College of Life Science, Sichuan University, Chengdu 610065, China

[5] Department of Microbiology, College of Resources, Sichuan Agricultural University, Chengdu 611130, China;

[#] Xiaolin Li and Lijuan Yan contributed equally to the work.

* correspondence: Xiaolin Li kerrylee_tw@sina.com

Wei Tan tanweichengdu@126.com

Bo Zhang bozhang5658@foxmail.com

Table S5 Hit genes more than 1% of NR species

Species	Hit number	Percentage (%)
Auricularia delicata TFB-10046 SS5	46727	42.18
Batrachochytrium dendrobatidis JAM81	4529	4.09
Rhizopus delemar RA 99-880	4203	3.79
Arthrobotrys oligospora ATCC 24927	3578	3.23
Dichomitus squalens LYAD-421 SS1	3385	3.06
Trichoderma virens Gv29-8	2885	2.60
Trichoderma atroviride IMI 206040	2033	1.84
Trichoderma reesei QM6a	1908	1.72
