# Supplementary material for: Transcriptional profiling of Auricularia cornea in selenium accumulation
Source: Sci Rep. 2019 Apr 4;9:5641. doi: 10.1038/s41598-019-42157-2 (PMC6449350; doi:10.1038/s41598-019-42157-2)
Supplement: Supplementary file 9 — Supplementary Table 6 [file 41598_2019_42157_MOESM9_ESM.pdf]

Transcriptional profiling of *Auricularia cornea* in selenium accumulation

Xiaolin Li[1#*], Lijuan Yan[2#], Qiang Li[3,4], Hao Tan[1], Jie Zhou[1], Renyun Miao[1], Lei Ye[1], Weihong Peng[1], Xiaoping Zhang[5], Wei Tan[1*], Bo Zhang[1*]

[1] Soil and Fertilizer Institute, Sichuan Academy of Agriculture Sciences, Chengdu 610066, China;

[2] Chair for Aquatic Geomicrobiology, Institute of Biodiversity, Friedrich Schiller University Jena, Jena, D-07743, Germany

[3] Biotechnology and Nuclear Technology Research Institute, Sichuan Academy of Agricultural Sciences, Chengdu 610061, China

[4] College of Life Science, Sichuan University, Chengdu 610065, China

[5] Department of Microbiology, College of Resources, Sichuan Agricultural University, Chengdu 611130, China;

[#] Xiaolin Li and Lijuan Yan contributed equally to the work.

* correspondence: Xiaolin Li kerrylee_tw@sina.com

Wei Tan tanweichengdu@126.com

Bo Zhang bozhang5658@foxmail.com

Table S6 Investigation of the top 10 GOSlim

Catergory	GO ID	Description	Number	Percentage (%)
biological_process	GO:0009987	cellular process	21794	10.21
biological_process	GO:0008152	metabolic process	20130	9.43
cellular_component	GO:0005623	cell	19858	9.30
molecular_function	GO:0003824	catalytic activity	19833	9.29
cellular_component	GO:0044464	cell part	19757	9.25
molecular_function	GO:0005488	binding	17519	8.20
biological_process	GO:0044699	single-organism process	15862	7.43
cellular_component	GO:0043226	organelle	13860	6.49
cellular_component	GO:0016020	membrane	8797	4.12
cellular_component	GO:0032991	macromolecular complex	8245	3.86
