# Supplementary material for: Transcriptional profiling of Auricularia cornea in selenium accumulation
Source: Sci Rep. 2019 Apr 4;9:5641. doi: 10.1038/s41598-019-42157-2 (PMC6449350; doi:10.1038/s41598-019-42157-2)
Supplement: Supplementary file 10 — Supplementary Table 7 [file 41598_2019_42157_MOESM10_ESM.pdf]

Transcriptional profiling of *Auricularia cornea* in selenium accumulation

Xiaolin Li[1#*], Lijuan Yan[2#], Qiang Li[3,4], Hao Tan[1], Jie Zhou[1], Renyun Miao[1], Lei Ye[1], Weihong Peng[1], Xiaoping Zhang[5], Wei Tan[1*], Bo Zhang[1*]

[1] Soil and Fertilizer Institute, Sichuan Academy of Agriculture Sciences, Chengdu 610066, China;

[2] Chair for Aquatic Geomicrobiology, Institute of Biodiversity, Friedrich Schiller University Jena, Jena,

D-07743, Germany

[3] Biotechnology and Nuclear Technology Research Institute, Sichuan Academy of Agricultural Sciences, Chengdu 610061, China

[4] College of Life Science, Sichuan University, Chengdu 610065, China

[5] Department of Microbiology, College of Resources, Sichuan Agricultural University, Chengdu 611130, China;

[#] Xiaolin Li and Lijuan Yan contributed equally to the work.

* correspondence: Xiaolin Li kerrylee_tw@sina.com

Wei Tan tanweichengdu@126.com

Bo Zhang bozhang5658@foxmail.com

Table S7 Investigation of unigenes within different KEGG types

KEGG type	KEGG Pathway	Unigene NO.	Percentage	Total percentage
Metabolism	Overview	352	6.15	
	Carbohydrate metabolism	419	7.32	
	Energy metabolism	323	5.65	
	Lipid metabolism	196	3.43	
	Nucleotide metabolism	175	3.06	
	Amino acid metabolism	329	5.75	
	Metabolism of other amino acids	111	1.94	41.57
	Glycan biosynthesis and metabolism	80	1.4	
	Metabolism of cofactors and vitamins	202	3.53	
	Metabolism of terpenoids and polyketides	45	0.79	
	Biosynthesis of other secondary metabolites	48	0.84	
	Xenobiotics biodegradation and metabolism	98	1.71	
	Enzyme families	0	0	
Genetic Information Processing	Transcription	247	4.32	
	Translation	751	13.13	
	Folding, sorting and degradation	382	6.68	26.51
	Replication and repair	136	2.38	
	RNA family	0	0	
Environmental Information Processing	Membrane transport	11	0.19	
	Signal transduction	375	6.55	6.76
	Signaling molecules and interaction	1	0.02	
Cellular Processes	Transport and catabolism	335	5.86	
	Cell motility	48	0.84	11.23
	Cell growth and death	194	3.39	

	Cellular commiunity	65	1.14	
Organismal Systems	Immune system	129	2.25	13.93
	Endocrine system	182	3.18	
	Circulatory system	82	1.43	
	Digestive system	66	1.15	
	Excretory system	68	1.19	
	Nervous system	126	2.2	
	Sensory system	26	0.45	
	Development	37	0.65	
	Aging	82	1.43	
	Environmental adaptation	0	0	
