# Supplementary material for: Transcriptional profiling of Auricularia cornea in selenium accumulation
Source: Sci Rep. 2019 Apr 4;9:5641. doi: 10.1038/s41598-019-42157-2 (PMC6449350; doi:10.1038/s41598-019-42157-2)
Supplement: Supplementary file 11 — Supplementary Table 8 [file 41598_2019_42157_MOESM11_ESM.pdf]

Transcriptional profiling of *Auricularia cornea* in selenium accumulation

Xiaolin Li[1#*], Lijuan Yan[2#], Qiang Li[3,4], Hao Tan[1], Jie Zhou[1], Renyun Miao[1], Lei Ye[1], Weihong Peng[1], Xiaoping Zhang[5], Wei Tan[1*], Bo Zhang[1*]

[1] Soil and Fertilizer Institute, Sichuan Academy of Agriculture Sciences, Chengdu 610066, China;

[2] Chair for Aquatic Geomicrobiology, Institute of Biodiversity, Friedrich Schiller University Jena, Jena, D-07743, Germany

[3] Biotechnology and Nuclear Technology Research Institute, Sichuan Academy of Agricultural Sciences, Chengdu 610061, China

[4] College of Life Science, Sichuan University, Chengdu 610065, China

[5] Department of Microbiology, College of Resources, Sichuan Agricultural University, Chengdu 611130, China;

[#] Xiaolin Li and Lijuan Yan contributed equally to the work.

* correspondence: Xiaolin Li kerrylee_tw@sina.com

Wei Tan tanweichengdu@126.com

Bo Zhang bozhang5658@foxmail.com

Table S8 Top 10 genes of each treatment

Treatment	id	baseMean	ACKb	A100b	FoldChange(A100b/ACKb)	log2FoldChange	pval	padj
ACKb	c108419_g1	264851	**376696**	153006	0.41	-1.30	0.46	1
	c215107_g1	120385	**175721**	65049	0.37	-1.43	0.43	1
	c69488_g1	114175	**162129**	66221	0.41	-1.29	0.46	1
	c215106_g1	91528	**132969**	50087	0.38	-1.41	0.43	1
	c76941_g1	88704	**128698**	48711	0.38	-1.40	0.44	1
	c76936_g1	84051	**122601**	45500	0.37	-1.43	0.44	1
	c98233_g1	68524	**98756**	38293	0.39	-1.37	0.45	1
	c76662_g1	87987	**84455**	91520	1.08	0.12	0.86	1
	c89761_g1	49616	**73043**	26189	0.36	-1.48	0.26	1
	c1414_g1	65344	**70235**	60453	0.86	-0.22	0.46	1
A100b	c108419_g1	264851	376696	**153006**	0.41	-1.30	0.46	1
	c76662_g1	87987	84455	**91520**	1.08	0.12	0.86	1
	c88275_g1	71981	54910	**89052**	1.62	0.70	0.17	1
	c107720_g2	73695	60858	**86532**	1.42	0.51	0.16	1
	c69488_g1	114175	162129	**66221**	0.41	-1.29	0.46	1
	c215107_g1	120385	175721	**65049**	0.37	-1.43	0.43	1
	c1414_g1	65344	70235	**60453**	0.86	-0.22	0.46	1
	c215106_g1	91528	132969	**50087**	0.38	-1.41	0.43	1
	c107428_g1	40206	30454	**49957**	1.64	0.71	0.06	1
	c105797_g4	39058	28431	**49685**	1.75	0.81	0.03	1
**Treatment**	**id**	**baseMean**	**ACKm**	**A100m**	**foldChange(A CKm/A100m)**	**log2FoldChange**	**pval**	**padj**
ACKm	c107720_g2	109823	**134143**	85503	1.57	0.65	0.31	1
	c77398_g1	93165	**128605**	57725	2.23	1.16	0.08	1
	c75117_g1	91821	**126662**	56980	2.22	1.15	0.08	1
	c105797_g4	48736	**65252**	32220	2.03	1.02	0.27	1
	c91050_g1	54484	**58234**	50735	1.15	0.20	0.64	1
	c107496_g3	42287	**49338**	35235	1.40	0.49	0.48	1
	c88275_g1	41556	**47483**	35629	1.33	0.41	0.57	1
	c105088_g1	33362	**46347**	20376	2.27	1.19	0.23	1

	c104952_g3	48514	**44623**	52405	0.85	-0.23	0.80	1
	c107939_g1	35148	**43413**	26883	1.61	0.69	0.29	1
A100m	c107720_g2	109823	134143	**85503**	1.57	0.65	0.31	1
	c77398_g1	93165	128605	**57725**	2.23	1.16	0.08	1
	c75117_g1	91821	126663	**56980**	2.22	1.15	0.08	1
	c104952_g3	48514	44623	**52405**	0.85	-0.23	0.80	1
	c108436_g2	36828	22516	**51140**	0.44	-1.18	0.26	1
	c91050_g1	54484	58234	**50735**	1.15	0.20	0.64	1
	c93327_g1	31530	16277	**46783**	0.35	-1.52	0.03	1
	c92768_g1	41023	40971	**41075**	1.00	-0.00	0.87	1
	c97717_g1	39651	41331	**37971**	1.09	0.12	0.81	1
	c83498_g1	25599	13237	**37961**	0.35	-1.52	0.03	1

Differentially expressed unigenes were screened according to Fold Change and P-value based on DESeq (Version 1.18.0) with the criteria |log2(FoldChange)| > 1 & p-value < 0.05. Abbreviations: *ACK* control group without selenium addition in the substrate; *A100* treatment group with 100 μg/g of selenium addition in the substrate; *m* at the mature stage; *b* at the budding stage.
