# Supplementary material for: Transcriptional profiling of Auricularia cornea in selenium accumulation
Source: Sci Rep. 2019 Apr 4;9:5641. doi: 10.1038/s41598-019-42157-2 (PMC6449350; doi:10.1038/s41598-019-42157-2)
Supplement: Supplementary file 12 — Supplementary Table 9 [file 41598_2019_42157_MOESM12_ESM.pdf]

Transcriptional profiling of *Auricularia cornea* in selenium accumulation

Xiaolin Li[1#*], Lijuan Yan[2#], Qiang Li[3,4], Hao Tan[1], Jie Zhou[1], Renyun Miao[1], Lei Ye[1], Weihong Peng[1], Xiaoping Zhang[5], Wei Tan[1*], Bo Zhang[1*]

[1] Soil and Fertilizer Institute, Sichuan Academy of Agriculture Sciences, Chengdu 610066, China;

[2] Chair for Aquatic Geomicrobiology, Institute of Biodiversity, Friedrich Schiller University Jena, Jena, D-07743, Germany

[3] Biotechnology and Nuclear Technology Research Institute, Sichuan Academy of Agricultural Sciences, Chengdu 610061, China

[4] College of Life Science, Sichuan University, Chengdu 610065, China

[5] Department of Microbiology, College of Resources, Sichuan Agricultural University, Chengdu 611130, China;

[#] Xiaolin Li and Lijuan Yan contributed equally to the work.

* correspondence: Xiaolin Li kerrylee_tw@sina.com

Wei Tan tanweichengdu@126.com

Bo Zhang bozhang5658@foxmail.com

Table S9 Primers designed for validation of gene expression profile in A. cornea transcriptome data

Gene ID	Primer ID	Primer sequences	Amplified products
c135295_g1	c135295_g1-F	AGAACACCGCTACAGGCTTC	122 bp
	c135295_g1-R	GACGAAGGTCCACGACTGTT	
c108476_g4	c108476_g4-F	AGAGCATTCCGACATCGCAT	200 bp
	c108476_g4-R	CTTCCAGTCCTTGACACGCT	
c102698_g4	c102698_g1-F	CTTAATTGGTGGCATCGCCG	209 bp
	c102698_g1-R	TGACCTCGAACACTTCGGTG	
c96467_g1	c96467_g1-F	CCGCCTATCAACGAGGAACA	126 bp
	c96467_g1-R	GGTGGACTGTTCGGTGAAGT	
RPL4(reference gene)	RPL4-F	GTCAACAAGGGCGTTCTCTT	151 bp
	RPL4-R	ACAGCGTCTTGAGGAAGGT	
